# A transdisciplinary approach to obesity and cancer: from cells to society

**DOI:** 10.1186/1753-6561-6-S3-O18

**Published:** 2012-06-01

**Authors:** Linda Nebeling

**Affiliations:** 1 National Cancer Institute, USA

## 

The increasing recognition of the complex, multidimensional relationship between excess adiposity and cancer risk, coupled with the high prevalence of obesity in the US, has motivated the scientific community to seek new research models and paradigms. Compared to traditional unidisciplinary research, transdisciplinary research is seen as having the potential to accelerate both discovery and its translation to practice, and eventually policy. In response, the National Cancer Institute (NCI) developed a centers grant mechanism in nutrition, energetics, and physical activity; referred to as the Transdisciplinary Research on Energetics and Cancer (TREC) Initiative (http://www.trecscience.org). These Centers foster collaboration among transdisciplinary teams of scientists with the goal of accelerating progress towards reducing cancer incidence, morbidity and mortality associated with obesity, low levels of physical activity and poor diet. This presentation will review key opportunities in transdisciplinary research, highlight lessons learned and note future challenges.

